# Closing Editorial: Mercury Cycling and Health Effects

**DOI:** 10.3390/toxics13100841

**Published:** 2025-10-02

**Authors:** José Vicente Elias Bernardi, Wanderley Rodrigues Bastos, Jurandir Rodrigues de Souza, Carlos José Sousa Passos

**Affiliations:** 1 Geostatistics and Geodesy Laboratory, UnB Planaltina College, University of Brasilia, Planaltina 73345-010, DF, Brazil; 2 Laboratório de Biogeoquímica Ambiental, Universidade Federal de Rondônia, Porto Velho 76815-800, RO, Brazil; bastoswr@unir.br; 3 Laboratório de Química Analítica e Ambiental, Instituto de Química, Universidade de Brasília, Brasília 70910-900, DF, Brazil; rodsouza@unb.br; 4 Faculty UnB at Planaltina (FUP/UnB) Planaltina, University of Brasília, Brasília 73345-010, DF, Brazil; cjpassos@unb.br

Mercury (Hg) is a persistent global contaminant that cycles among the atmosphere, rivers, oceans, soils, and biota. In aquatic ecosystems, inorganic forms are photochemically, and preferably, microbially transformed into methylmercury (MeHg), which bioaccumulates in fish and biomagnifies up food webs—the dominant human exposure route in most populations, especially those most isolated from urban areas. The World Health Organization identifies mercury as one of the top chemicals of major public-health concern; MeHg is a well-established neurotoxicant with vulnerability during fetal and early-life development [1].

Global inventories attribute the largest share of anthropogenic Hg emissions to artisanal and small-scale gold mining (ASGM), followed by fossil-fuel combustion and certain industrial processes. Once emitted, atmospheric Hg undergoes long-range transport and hemispheric redistribution before deposition, where it can be emitted or methylated depending on the biogeochemical conditions. The Global Mercury Assessment (2018) and its technical background syntheses remain foundational references for quantifying sources, environmental transport, and regional hotspots [2,3].

The Minamata Convention on Mercury—adopted in 2013 and in force since 2017—establishes binding controls on a wide spectrum of Hg sources including primary mining, product manufacture and trade, emissions to air and releases to aquatic ecosystems, storage and waste, and informal sectors such as ASGM. The Convention has catalyzed national action plans, phase-outs, and technology upgrades; however, implementation gaps persist, and many countries face capacity constraints in monitoring, enforcement, and risk communication [4].

From a health perspective, elemental Hg vapor (Hg^0^) is efficiently absorbed via inhalation and is oxidized to inorganic Hg, with the kidneys and brain as principal deposition sites; chronic exposure can lead to neurobehavioral changes and renal effects. The U.S. EPA’s Integrated Risk Information System (IRIS) sets a chronic inhalation reference concentration (RfC) of 0.3 μg·m^−3^ for Hg^0^, derived from human occupational studies of subtle neurobehavioral endpoints [5], while the ATSDR Toxicological Profile synthesizes evidence across exposure routes, toxicokinetics, and susceptible populations [6].

Risk management and governance must also account for the benefits of fish consumption—especially long-chain omega-3 fatty acids and high-quality protein—against MeHg risk. The 2023 Joint FAO/WHO Expert Consultation provides an updated framework for balancing the nutritional benefits of fish with contaminant risks, supporting risk-communication approaches that are species-, population-, and context-specific [7].

Against this backdrop, this Special Issue brings together complementary perspectives on MeHg exposure and health: multi-decadal biomonitoring of consumed fish, trophic-position indicators using Hg, a systematic review of regional contamination patterns in biota and humans, maternal exposure during pregnancy, mechanistic links between prenatal MeHg and neurodevelopment, and basin-scale modeling of Hg dynamics and consumption risk. Together, these contributions illuminate data gaps, methodological needs, and policy opportunities that can inform future surveillance, intervention design, and Minamata Convention implementation.

Occupational and urban inhalation exposures. Most papers emphasize diet-related MeHg exposures; inhalation of elemental Hg^0^ in urban/industrial or WWTP settings is under-represented, thus there is a need to complement dietary pathways and reflect diverse global contexts.

Intervention and risk-communication studies. Stronger evaluation of advisories (e.g., fish-switching strategies), community co-design, and policy impact tracking (pre/post interventions) are needed, especially for indigenous communities in hotspot basins.

Longitudinal epidemiological studies. Extend from cross-sectional biomarkers (e.g., hair in pregnancy) to prospective cohorts linking exposure, molecular markers (omics, epigenetics), and neurocognitive outcomes (infancy → adolescence).

Food-web integration beyond fish. Vegetables, fruits, rice, and bushmeat in Hg-impacted regions remain understudied; coupled isotope/Hg speciation could refine trophic and source apportionment.

Comparability and QA/QC. Harmonized analytical methods, reporting units, detection limits, and uncertainty; wider use of CRMs and inter-lab comparisons to support meta-analysis and regulatory use.

Model-data fusion. Broaden SERAFM-like modeling with uncertainty quantification, remote sensing (land-use/deforestation), and climate variability; validate with targeted field campaigns.

Geographic balance. While the Amazon is appropriately prominent, future calls should solicit studies from Africa, SE Asia, and Arctic/sub-Arctic systems to generalize the findings.

## Data Availability

No new data were created or analyzed in this study. Data sharing is not applicable to this article.

## References

[1] World Health Organization (WHO) (2024). Mercury and Health—Fact Sheet. https://www.who.int/news-room/fact-sheets/detail/mercury-and-health.

[2] United Nations Environment Programme (UNEP) (2019). Global Mercury Assessment 2018.

[3] AMAP/UN Environment (2019). Technical Background Report to the Global Mercury Assessment 2018.

[4] Minamata Convention on Mercury (2017). Text and Annexes.

[5] U.S. Environmental Protection Agency (EPA) Integrated Risk Information System (IRIS): Mercury, Elemental (CASRN 7439-97-6). Reference Concentration (RfC) = 0.3 μg/m^3^. https://iris.epa.gov.

[6] Agency for Toxic Substances and Disease Registry (ATSDR) (2022). Toxicological Profile for Mercury.

[7] FAO/WHO (2023). Joint FAO/WHO Expert Consultation on the Risks and Benefits of Fish Consumption—Updated Framework.

